# The New Zealand Indices of Multiple Deprivation (IMD): A new suite of indicators for social and health research in Aotearoa, New Zealand

**DOI:** 10.1371/journal.pone.0181260

**Published:** 2017-08-03

**Authors:** Daniel John Exeter, Jinfeng Zhao, Sue Crengle, Arier Lee, Michael Browne

**Affiliations:** Section of Epidemiology & Biostatistics, School of Population Health, The University of Auckland, Auckland, New Zealand; Ege Universitesi, TURKEY

## Abstract

For the past 20 years, the New Zealand Deprivation Index (NZDep) has been the universal measure of area-based social circumstances for New Zealand (NZ) and often the key social determinant used in population health and social research. This paper presents the first theoretical and methodological shift in the measurement of area deprivation in New Zealand since the 1990s and describes the development of the New Zealand Index of Multiple Deprivation (IMD).

We briefly describe the development of Data Zones, an intermediary geographical scale, before outlining the development of the New Zealand Index of Multiple Deprivation (IMD), which uses routine datasets and methods comparable to current international deprivation indices. We identified 28 indicators of deprivation from national health, social development, taxation, education, police databases, geospatial data providers and the 2013 Census, all of which represented seven Domains of deprivation: Employment; Income; Crime; Housing; Health; Education; and Geographical Access. The IMD is the combination of these seven Domains. The Domains may be used individually or in combination, to explore the geography of deprivation and its association with a given health or social outcome.

Geographic variations in the distribution of the IMD and its Domains were found among the District Health Boards in NZ, suggesting that factors underpinning overall deprivation are inconsistent across the country. With the exception of the Access Domain, the IMD and its Domains were statistically and moderately-to-strongly associated with both smoking rates and household poverty.

The IMD provides a more nuanced view of area deprivation circumstances in Aotearoa NZ. Our vision is for the IMD and the Data Zones to be widely used to inform research, policy and resource allocation projects, providing a better measurement of area deprivation in NZ, improved outcomes for Māori, and a more consistent approach to reporting and monitoring the social climate of NZ.

## Introduction

There is an unequivocal graded association between area-based deprivation, health and social outcomes in New Zealand [1–4] and elsewhere.[5–10] The accurate measurement of area-based socioeconomic deprivation is vital for planning and for ensuring that resource allocation formulae are equitable and target areas with the highest levels of need. Indeed, area-based measures of deprivation have been integral in research, planning and advocacy initiatives for almost 50 years. For example, in the United Kingdom (UK) research into social and material deprivation emerged following the release of 1971 Census data for small areas. [11], [12]

During the 1980s Townsend made the theoretical distinction between ‘poverty’ and ‘deprivation’ using data from the 1981 UK Census.[13] Townsend defined deprivation as “a state of observable and demonstrable disadvantage relative to the local community or the wider society or nation to which an individual, family or group belongs”. [13] Moreover, he argued that deprivation comprises material and social aspects, in which material deprivation referred to the goods, services, resources, amenities, physical and living environments, while social deprivation comprised the roles, relationships, functions, customs, rights and responsibilities of membership of society and its subgroups.[13] Within this context, individuals can therefore experience multiple forms of deprivation and they may have a cumulative effect. Townsend’s definition established the foundations upon which subsequent research into area deprivation (globally) have been based.

Townsend developed an index that used four Census-derived indicators of deprivation (unemployment, household overcrowding, non-home ownership and non-car ownership). Each indicator was selected for its theoretical ability to measure aspects of social or material deprivation, but were also predicated on the availability of 1981 Census data. Similarly, Carstairs and Morris [6] developed an index for Scotland’s Postcode Sectors, which were similar in scale to Census Wards in England and Wales. Their index also comprised four variables derived from the 1981 UK Census (proportions of male unemployment, lack of car ownership, low social class, and household overcrowding). Both indices were used extensively to better understand health inequalities in the UK. The Townsend Index was used to demonstrate that the widening inequalities in the UK during the 1970s and 1980s were real and worse than estimated by the Black Report. [14]

The UK government developed its own Index of Local Conditions (1991) and the Index of Local Deprivation (ILD) in 1998, which were produced at three spatial scales based on 1991 Census boundaries: Enumeration District (ED, approximately 101,000; 420 people on average); Census Ward (approximately 8,620; 5,000 people); and Local Authority District (354 in 1998; 122,000 people). At the Local Authority District scale, the ILD included 12 indicators, while the ED and Census Ward versions used five and six indicators respectively. [15] Concerns regarding the Government’s dependence on decennial Census data for guiding investments in deprived communities led to some early use of routine health and social data collected by government departments and agencies. Building on the ILD, the first Indices of Multiple Deprivation (IMD) were developed for England in 2000 by the *Department of the Environment*, Transport and the Regions (*DETR*). They used routinely collected data, incorporating direct, (employment, housing, geographic access) and indirect (income, health) measures of the causes and/or consequences of deprivation and were used by the Government to allocate billions of pounds of resources.

### Measuring deprivation in New Zealand

In 1985, Reinken et al. [16] first attempted to measure area deprivation in New Zealand using data from the 1981 Census with Census Area Units (CAUs) as the unit of analysis. The purpose of their study was to “identify neighbourhoods which can be presumed to have a special need for preventive health services” (Reinken et al, 1985: p42). They identified 17 items from the Census representing demographic (e.g. age, ethnicity, marital status) and socio-economic (e.g. “blue collar” employees, receiving a means-tested benefit, seeking employment, home ownership, average weekly rental and car ownership) factors to measure deprivation. They used Principal Components Analysis to identify the key factors of deprivation and how they differed by gender, for urban or rural areas, and the effect that excluding age-specific variables (e.g. dependent populations) had on the final index.

Following the 1991 Census, the New Zealand Index of Deprivation (NZDep) was developed in response to calls for a tool to assist with needs-based resource allocation. Whereas Reinken et al’s work was based on CAUs (average population 2,000 people), NZDep was created using Meshblock (MBs) (average population 100 people). NZDep used national Census data, and was based on international deprivation research. It was conceived with three purposes in mind: resource allocation, community advocacy and research. [17] The methodology for NZDep1991, which was broadly consistent with Reinken’s work, combined nine indicators of social and material deprivation from the Census using Principal Components Analysis. Where Reinken had apportioned CAUs into 7 categories of deprivation, NZDep’s standardised Principal Components score was classified into Deciles, with Decile 1 representing the 10% least deprived small areas (MBs) in New Zealand and Decile 10 representing the 10% most deprived areas in the country. NZDep has become widely used in health [18–20] and social [21, 22] research in addition to being a key variable in funding allocation models. [23–25]

Despite their popularity in population health research, Census-based indices have their limitations. As most countries hold their national Census every five or 10 years, the indices become quickly outdated, especially in communities that undergo substantial population and social change through urban regeneration programmes, for example. One limitation of NZDep is that researchers are unable to easily deconstruct the index and use some of its nine variables independently to determine for example, the strength of an association between a given health outcome and different categories of deprivation. Furthermore, most Census-based indices are based on assumptions of how particular variables represent material deprivation. Consider the inclusion of motor vehicle ownership in the NZDep, Carstairs and Townsend indices. Motor vehicle ownership may measure a household’s ability to access material resources, however there is evidence that area-based deprivation indices that include car ownership variables are poor indicators of deprivation in rural areas, since car ownership is a necessity of rural life.[26] In addition, city dwellers require cars less often if they live in close proximity to their workplace and have good access public transport. Given that the proportion of households in New Zealand without access to a motor vehicle decreased from 12% to 8% between the 1991 and 2006, one may also argue that its overall utility as a measure of deprivation is becoming more limited. Similarly, the near-universal access to telephones in 2006 (94–99%), [27] reduced the significance of this indicator when measuring disadvantage and led NZDep2013 to capture households without access to the Internet instead. [28]

In response to these limitations, and supported by an increasing ability to access routine administrative data sources, a new deprivation index has been developed for New Zealand that is underpinned by Townsend’s [13] definition of deprivation and enhanced by Noble et al. [29, 30] who state that “multiple deprivation is thus not some separate form of deprivation. It is simply a combination of more specific forms of deprivation, which themselves can be more or less directly measurable”. This research contributes to the wider literature which combines indicators of a particular type of deprivation into a ‘Domain’ of deprivation and then combines those Domains into an overall measure of multiple deprivation at the small area level.[9, 30, 31]

In this paper, the development of a customised geographical boundary file known as Data Zones (DZs) is outlined, before the 28 indicators chosen to represent seven Domains of deprivation and the creation of the New Zealand Index of Multiple Deprivation (IMD) are described. We then validate the association between the IMD and its Domains against smoking rates and household-level poverty, before exploring geographic variations in the IMD and its Domains for the 20 District Health Boards (DHBs) across NZ.

## Data and methods

### Ethical approval

This study was given ethical approval by the Chairperson of the Northern X Regional Ethics Committee on 24 August 2011, with ongoing approval granted by the New Zealand Health and Disability Ethics Committees (Ref: NTX/11/EXP/190).

### Designing the geographical base—Data Zones

The availability of cross-tabulated data for more than one dimension (such as a population by age and ethnicity) is limited at the meshblock (MB) level, due to Statistics NZ’s disclosure and confidentiality rules, so there was a need for the development of a standard neighbourhood-level geography, optimised for social and health research. We extended the methodology outlined by Zhao and Exeter [32] to create a customised geographical base known as Data Zones (DZ) using 2013 MB boundaries (the smallest Census area output) as building blocks. For a detailed account of the DZ construction, readers are directed to Zhao and Exeter (32). In brief, we constructed 5,958 DZs with an average population of 712. There were 46,629 MBs in 2013 that had populations ranging from 0 to 1,899 with a median of 78. We excluded 708 MBs which were either oceanic, water-inlets or lakes and had a combined population of 738 residents, from the 4,242,132 usual resident population of NZ in 2013. We used 45,921 MBs to construct the 5,958 DZs using six criteria commonly associated with geographic zone design for health and social research (geographic contiguity, population equality, respecting administrative boundaries, respecting physical barriers, internal socioeconomic homogeneity and compactness). With the exception of one ‘small’ DZ, representing all of Stewart Island (total population of 384) and 10 ‘large’ DZs with populations between 1,381 and 1,899 (mostly comprising a single MB) the population of the DZs ranged from 501 to 999. While Zhao and Exeter[32] nested their ‘Lower Zones’ for Auckland within Census Wards, in this study and following consultation with Statistics NZ’s geospatial team, the DZs are nested within CAUs wherever possible (93.3%). In addition, the DZs nest within higher geographical units such as Electoral Districts, Territorial Authorities, District Health Boards and Regions. One strength of these DZs is that they are independent of the administrative units used by different government agencies (e.g. Police Districts, School Zones), which may change over time, and thus represent a neutral geographical basis for facilitating data sharing. DZs were not intended to reflect the true extent of actual communities; rather they are an intermediate geography between MB and CAU that facilitates small-area analyses of health and social data at a scale small enough to be statistically robust, while also conveying a sense of neighbourhood. [32]

### Indicator selection and Domain development

Following a literature review, a hui (meeting) with Māori and non-Māori stakeholders, and discussions with topic-experts and data managers at the Ministries of Education, Health, Social Development, Inland Revenue, Housing NZ Corporation, NZ Police, other local and international researchers, we conducted a comprehensive stocktake of potential indicators and data sources, focussing on items that measured different forms of deprivation as directly as possible. Potential indicators discussed included measures of income, employment, crime, housing, health and education, in addition to measures of wealth, literacy, community connectedness, pollution, access to basic amenities (e.g. schools, pharmacies, supermarkets) and access to social hazards (e.g. problem gambling, alcohol outlets). We also discussed notions of indigeneity, racism and Māori development, causes and consequences of deprivation, under reporting of certain criminal offence types, and measuring the utilisation of services. As a result of this stocktake, we identified seven Domains of deprivation relevant to New Zealand: Employment; Income; Education; Housing; Health; Crime and Geographical Access.

Consistent with previous deprivation research, [6, 9, 13, 30, 33–35] indicators were selected for their theoretical ability to measure a particular aspect of relative deprivation and if they: were nationally available at the street address or small area (MB) level; measured major characteristics of a given aspect of deprivation; were not conditions with a limited geographic or demographic exposure; reflected circumstances of relevance to New Zealand; were up-to-date and easily updatable and were statistically robust. [9, 30, 33]

**Fig 1:**
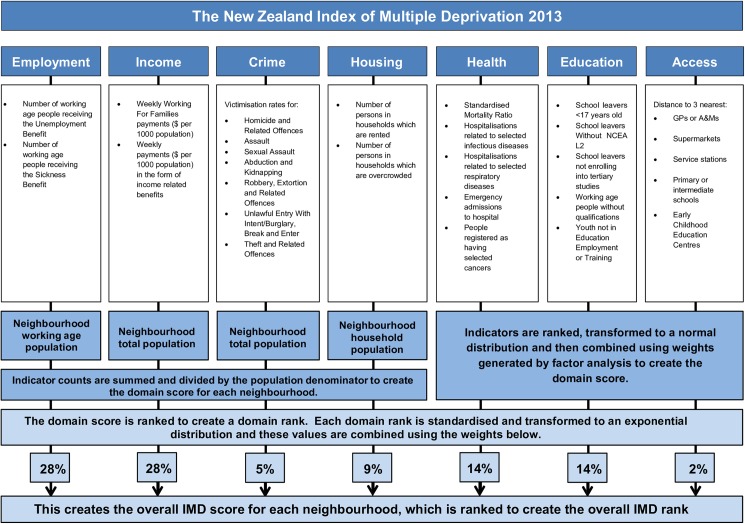
Developing the IMD: An overview. Adapted from Fig 2 SIMD 2012 Methodology, in Scottish Index of Multiple Deprivation 2012. Edinburgh: Scottish Government (Crown Copyright 2012, See S1 Fig). [36] Reproduced with Permission (see S1 File)

Fig 1 provides a summary of the 28 indicators that met the inclusion criteria and their allocation into Domains. For each indicator, we obtained data for 2013, or as close to 2013 as possible, and we used 2013 Census counts as denominators for all but three Education indicators, for which counts of school leavers were used. For example, the Employment Domain includes the proportion of working age (15–64) adults who were receiving the Unemployment Benefit or the Sickness Benefit in the last week of March 2013. This period was selected as it was the Ministry of Social Development’s reporting period closest to the 2013 Census night. By contrast, the only data available for the Crime Domain were for the 11 months between 1 July 2014 to 31 May 2015, and we used the total population from the 2013 Census as the denominator. All statistical procedures were conducted in SAS version 9.4, except for measures of proximity to basic amenities used in the Access Domain, which were calculated in ArcGIS 10.3.0.

Our Employment Domain measures those individuals who are willing to work but are unable to do so through unemployment or sickness. This Domain comprises two indicators using data from the Ministry of Social Development (MSD): the working age population receiving the Unemployment Benefit and the working age population receiving a Sickness Benefit on the last week of March 2013. As claimants can only receive one of these benefits at any given time, these two indicators are mutually exclusive. The purpose of these indicators is to measure the degree to which working age people are excluded from the employment world, but they do not capture aspects of hidden unemployment such as those who are seeking work but not registered with Work and Income New Zealand (a subsidiary of MSD). Counts for the working age population receiving the Unemployment Benefit or the Sickness Benefit in each DZ were totalled and divided by the working age population to form an Employment Domain score, which was then ranked from 1 to 5,958 in order of increasing deprivation.

There were two indicators in the Income Domain. These capture the extent of income deprivation in a neighbourhood by measuring the financial assistance provided by the State in the last week of March 2013 to those whose income was deemed insufficient. One indicator measures payments from the MSD in the form of selected income-tested benefits and Working for Families (WFF) Tax Credits paid to beneficiaries. The other indicator measures payments by Inland Revenue (IR) including WFF Tax Credits paid to working people, Child Tax Credits and Paid Parental Leave. Data for the IR indicator were extracted from Statistics New Zealand’s Integrated Data Infrastructure (IDI). The IDI is a data repository comprising anonymised, individual-level data routinely collected by government agencies, in addition to surveys conducted by Statistics NZ including the 2013 Census, and non-government organisations.[37] In this study, the total amount paid in each DZ in the various forms of income support was divided by the total population to form an Income Domain score, which was then ranked from 1 to 5,958 in order of increasing deprivation.

In consultation with the New Zealand Police, we used data from the new Recorded Crime Victimisation Statistics (RCVS) to construct our Crime Domain. RCVS data for 236,277 victimisations for the period between 1 July 2014 and 31 May 2015 were extracted from the IDI. In 2015 Statistics New Zealand [38] recommended that victimisations should be counted after 30 days of investigation, as many investigations are still active at seven days. Therefore, we extracted counts of victimisations after 30 days of investigation for the seven major offence types captured by the RCVS: Homicide and Related Offences; Assault; Sexual Assault; Abduction and Kidnapping; Robbery, Extortion and Related Offences; Unlawful Entry with Intent/Burglary, Break and Enter; Theft and Related Offences. The majority (54.6%) of victimisations related to Theft, and together with Unlawful Entry (24.4%) and Assault (17.5%) accounted for 94% of all victimisations reported in the 11 month period.[38] We allocated victimisations to DZs using the MB of the scene of an offence, calculated a victimisation rate per 1,000 population using 2013 Census data, and ranked the scores in order of increasing deprivation.

Potential indicators for the Housing Domain, such as measures of the age of the housing stock, housing quality, affordability, applications to the Tenancy Tribunal, and households whose landlord is either the State-owned Housing NZ Corporation (HNZC) or a Local Authority were explored but rejected for a variety of reasons. In most cases the data were not available in a consistent form for the entire country. Therefore, the Housing Domain comprises two indicators obtained from the 2013 Census: the proportion of the population living in overcrowded households and the proportion of the population living in rented accommodation. Consistent with Statistics NZs protocols, we used the Canadian National Occupancy Standard (CNOS) [39] in which overcrowding was defined as households requiring one or more extra bedrooms to adequately accommodate occupants, based on the relationships between household members and their ages. The indicator representing rented accommodation included renting from HNZC and other social housing providers, as well as from private landlords. The two indicators were combined using weights consistent with the deprivation literature (in which overcrowding is typically weighted higher than other housing-related measures [40–42]), and we tested their correlations with three known deprivation-related measures–the IMD Employment and Income Domains and smoking rates. We found that the household overcrowding variable was consistently more closely related to these deprivation measures than the proportion of the population living in rented dwellings. We therefore normalised the overcrowding and renting percentages so they were on the same scale of 0 to 1 and combined them with 60% and 40% weights respectively. As factor analysis on two items is not recommended, we tested three different methods of normalisation (min-max normalisation, normalisation using the Blom method, and using the ranks directly) for sensitivity analyses. The resulting housing domain ranks were highly correlated, with the Spearman correlation coefficients ranging from 0.981 to 0.998. Given the consistency of these results, we selected the min-max normalisation method and combined the two indicators with 60% / 40% weighting to form the Housing Domain score, which was then ranked in order of increasing deprivation. It is important to note that this domain will be less responsive to changes over time as the indicators cannot be updated until after the 2018 Census.

After exploring many potential health indicators with clinicians, colleagues, and Ministry of Health analysts, the Health Domain comprises five indicators that use data obtained from the Ministry of Health to identify areas with a higher than expected level of ill health or mortality for the age profile of the population. In order to average any anomalies associated with uncharacteristic patterns, we obtained counts of mortality events between 2011 and 2014, acute hospitalisation events per DZ between 2012 and 2014, and cancer registrations between 2010 and 2014. The different time periods reflect the period closest to the 2013 Census year that captures sufficient data points, and the availability of different data sets from the Ministry of Health.

While the Standardised Mortality Ratio (SMR) measures the observed versus expected number of deaths across all ages in a given DZ, the remaining indicators focus on admissions to an emergency department, hospitalisations for infectious or respiratory diseases and cancer registrations for which the primary diagnosis was for illnesses that have a known social gradient. [e.g. 18, 43] To create the overall Health Domain score, the five indicator scores were ranked and transformed to a normal distribution, to prevent outliers having a disproportionate effect on the overall scores. The transformed ranks were combined using weights based on standardised scoring coefficients generated by factor analysis using the maximum likelihood method (Table 1), and the resulting scores were then ranked in order of increasing deprivation.

**Table 1:** Weights for ranked health indicators in the Health Domain.

Health indicators	Weight
Registrations for cancers with a social gradient	0.04
Standardised Mortality Ratio	0.08
Acute hospitalisations related to infectious diseases with a social gradient	0.19
Acute hospitalisations related to respiratory diseases with a social gradient	0.28
Emergency admissions to hospital	0.42

The Education Domain has five indicators, three of which were extracted from the IDI and measure the proportions of school leavers who left before they were 17 years old, without the equivalent of NCEA Level 2, and/or did not enrol in any level of tertiary studies within 3 years of leaving school. The other two indicators use 2013 Census data to measure the proportion of youth aged 15–24 not in education, employment, or training (NEET) to capture youth disengagement, and the proportion of the working age population without a formal qualification, to reflect the assertion that educational attainment influences individuals over their life-course. To create the overall domain score, the five indicator scores were ranked and transformed to a normal distribution to prevent outliers having a disproportionate effect on the overall scores. The transformed ranks were combined using weights based on standardised scoring coefficients generated by factor analysis (Table 2).

**Table 2:** Weights of ranked education indicators in the Education Domain.

Education indicators	Weight
School leavers not transitioning to tertiary studies	0.06
Youth not in Education, Employment, or Training	0.13
School leavers younger than 17 years old	0.25
Working age people 15–64 with no qualifications	0.26
School leavers with less than NCEA Level 2	0.30

We developed the Access Domain to represent the cost and inconvenience of travelling to access basic amenities. We obtained the geographic co-ordinates (x,y) of facilities including primary health care providers, supermarkets, service stations, early-childhood centres, and primary and intermediate schools from a range of data suppliers. We conducted spatial validation using Google Maps and made direct contact where necessary. Comparable research internationally calculated the travel time to the closest facility separately for both public and private transport. However, as New Zealand lacks a comprehensive national public transport system and evidence suggests the closest facility is not always the service utilised [44, 45], we opted to measure the distance to the nearest three localities of a given facility. To create the overall Domain score, the five indicator scores were ranked and transformed to a normal distribution to prevent outliers having a disproportionate effect on the overall scores. The transformed ranks were combined using weights based on standardised scoring coefficients generated by factor analysis, as shown in Table 3.

**Table 3:** Weights of ranked access indicators in the Access Domain.

Access Indicators	Weight
Early Childhood Education Centre	0.15
School for Years 1 to 8	0.15
Supermarket	0.20
Petrol station	0.23
GP or Accident and Emergency Clinic	0.26

### Developing the New Zealand Index of Multiple Deprivation (IMD)

To create the New Zealand Index of Multiple Deprivation (IMD), the Domain-specific scores for each DZ were ranked in order of increasing deprivation, transformed to an exponential distribution, and combined using the Domain weights shown in Fig 1. While the transformed Domain score had a range from 0 to 100, the constant in the exponential transformation formula ensured that approximately 10% of the DZs had a score of greater than 50.[30] The skewness in the exponential distribution reduces the extent to which high deprivation in one domain cancels out low deprivation in another domain. The IMD and each of its seven Domains were ranked in ascending order, with the most deprived DZ ranked at 5,958. If two or more DZs had the same score, they received a rank equal to the mean of the corresponding rank of the tied scores.

Consistent with previous research,[30, 46] the weights used to combine Domains into the overall IMD were driven by the literature on multiple deprivation. Given Townsend’s [13: p.131] observation that although ‘people experiencing some form of deprivation may not all have low income, people experiencing multiple or single but very severe forms of deprivation are in almost every instance likely to have very little income and little or no other resources’, the Income and Employment (the means to generate income) Domains have the most weight at 28% each. The Health and Education Domains have the second-highest weights (14%), followed by Housing (9%), and Crime (5%). The Access Domain has the lowest weight (2%) because access to basic services in NZ is negatively associated with deprivation, except for rural dwellers.[47] The ranks were further classified into Quintiles and Deciles to facilitate use of the IMD and its Domains in research and policy.

## Results

### Correlations between the IMD and its Domains at the data zone level

We used Spearman’s Rank Correlations, ρ, to re-confirm the strength of associations between the IMD and its Domains (Table 4) at the DZ level. All of the correlations are at least moderately or highly statistically significant (p<0.001), except the weak correlations with the Access Domain. The Income and Employment Domains were very strongly associated with IMD (ρ = 0.95 and 0.93 respectively). Similarly, the Health (ρ = 0.82), Education (ρ = 0.81) and Housing (ρ = 0.78) Domain ranks were strongly correlated with IMD ranks, while the Crime Domain was only moderately associated with IMD ranks (ρ = 0.60). The Access Domain (ρ = -0.29) was weakly and negatively associated with IMD, and raised considerable questions about its inclusion in the IMD. After debate it was decided to retain the Access Domain because in rural areas, poor access to basic amenities is a disadvantage. For example, we classified each DZ as urban or rural based on Statistics New Zealand’s Urban Rural Profile.[48] For the overall IMD, there was a general trend in which the proportion of DZs in rural areas decreased with increasing level of deprivation. In Decile 1 areas, 15% of DZs were rural, while in Decile 10 (most deprived), only 5% of DZs were rural. However 98% of the DZs in the most Access deprived Decile were rural, and no DZs were classified as rural below Decile 7 in the Access Domain.

**Table 4:** Correlations between the IMD, its Domains, with rates of smoking and household poverty.

Spearman Correlation Coefficients, N = 5,958								
	IMD	Employment	Income	Crime	Housing	Health	Education	Access
**IMD**	**1**							
**Employment**	0.93	**1**						
**Income**	0.95	0.86	**1**					
**Crime**	0.61	0.53	0.50	**1**				
**Housing**	0.77	0.68	0.70	0.63	**1**			
**Health**	0.82	0.72	0.76	0.47	0.62	**1**		
**Education**	0.81	0.68	0.78	0.37	0.47	0.60	**1**	
**Access**	-0.29	-0.27	-0.27	-0.46	-0.55	-0.34	0.01^*^	**1**
**Regular smokers**	0.81	0.71	0.79	0.42	0.56	0.60	0.83	-0.02^^^
**Households living in poverty**^**~**^	0.80	0.72	0.77	0.46	0.62	0.57	0.70	-0.19

^*^p = 0.36

^^^p = 0.13, all others p < 0.001

^~^ households earning less than 60% of the median Jensen Equivalised Annual Household Income (JEAHI)

The Income Domain’s strongest association is with Employment, while Crime’s strongest association is with Housing. Education and Health are strongly associated with the Income Domain, while the weakest associations are typically with the Access Domain. The correlations between Access and the Income, Employment and Health Domains (-0.26 to -0.35) are reasonably weak; associations between Access and Crime (-0.46) and Housing (-0.55) are moderate, but the association between Access and the Education Domain is not statistically significant (0.01, p = 0.36). In Table 4, we demonstrated the association between the IMD and its seven Domains against the ranked proportion of smokers and households earning less than 60% of the Jensen Equivalised Annual Household Income (JEAHI: ‘household poverty’) per DZ, using data from the 2013 Census to test the predictive and concurrent validity of the IMD respectively. While tobacco smoking has reduced substantially in New Zealand over recent decades, existing indices of occupational social class [49] and area-deprivation [28] have found that a strong socio-economic gradient persists. Table 4 demonstrates a very strong, positive and statistically significant (p<0.001) correlation between smoking rates and the overall IMD (0.81), and the Education (0.83), Income (0.79), and Employment (0.71) Domains. Moderate associations between smoking rates and the Health, Housing and Crime Domains are consistent with our theoretical assumptions that these three Domains are important determinants of area deprivation but should attract lower weights in the overall IMD. The IMD is also strongly correlated with the number of households earning less than 60% of the median (JEAHI)–a validated measure of household-level poverty, [50, 51] at the DZ level. Overall the results for the household poverty measure were consistent with the correlations reported for smoking. While smoking was most strongly correlated with the Education Domain, household poverty was unsurprisingly most strongly associated with the Income Domain, and was more strongly correlated with the Housing, Crime and Access Domains than smoking.

Given the IMD comprises an Income Domain–albeit based on income support payments to individuals rather than household income per se–one may argue that there is a degree of circularity when measuring the correlation between household poverty and the IMD or the Income Domain. In response to this, we created seven additional IMDs, each one excluding one of the 7 domains. The benefit of the IMD is the ease with which Domains can be excluded or included, depending on the topic under investigation. In the household poverty example above, we used the IMD (No Income) index, and found a correlation of 0.78, marginally less than reported for the overall IMD. Similarly, the Spearman Rank correlation between the IMD (No Health) index and smoking was 0.83, slightly higher than we found for the IMD and smoking.

## Geographical variations in multiple deprivation in New Zealand

**Fig 2:**
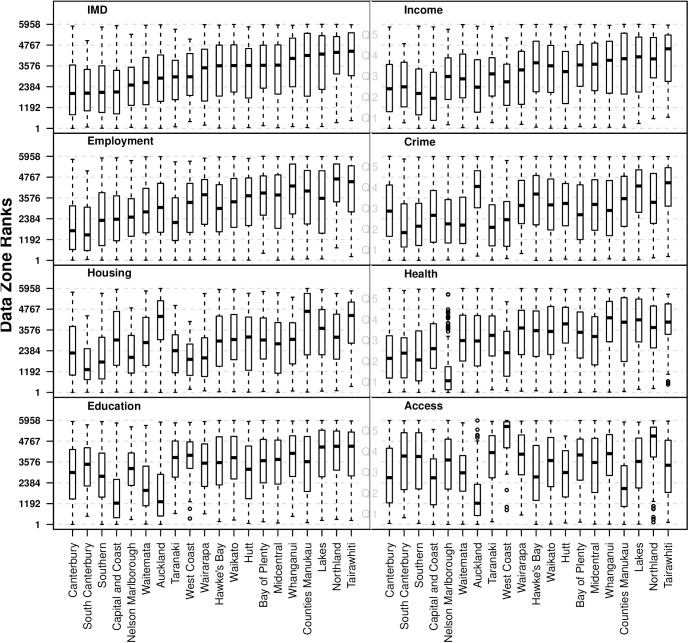
The distribution of (A) the IMD and its Domains (B-H), ordered by median IMD rank.

Fig 2 demonstrated the distribution of IMD ranks by District Health Board. The 20 territorial entities responsible for delivering health services in NZ are ordered by the median IMD rank in ascending levels of deprivation. For clarity, we refer here to the distributions in relation to deprivation Quintiles, with the least deprived Quintile (Q1) ranked 1 and the most deprived (Q5) ranked 5,958. Fig 2A shows that the Canterbury DHB had the lowest median IMD rank (2000), while Tairawhiti had the highest median IMD rank (4415). All DHBs have DZs in each of the IMD Quintiles of deprivation. However, as one might expect, the 10 DHBs with the lowest median IMD ranks are moderately skewed toward the least deprived IMD ranks, while those DHBs with higher medians are more skewed toward increasing levels of deprivation.

The boxplots shown in Fig 2B–2H, are also ordered according to increasing median IMD rank, and highlight the variations in Domains of deprivation within DHBs. These boxplots show that the Capital and Coast DHB (in Wellington, NZ’s Capital City) has among the lowest levels of Income and Education deprivation; Nelson/Marlborough DHB has by far the lowest Health deprivation; and the South Canterbury and Canterbury DHBs have the lowest employment deprivation. In contrast, the Auckland, Counties Manukau, and Tairawhiti DHBs have the worst Housing deprivation, and the Auckland, Lakes, and Tairawhiti DHBs have the most Crime deprivation. West Coast and Northland are the DHBs with the worst Access deprivation, while the Auckland DHB has the best.

The reader is invited to explore the geography of the IMD and its Domains using our interactive online atlases available at http://www.fmhs.auckland.ac.nz/imd.html.

## Discussion

This is the first national study in New Zealand to use routine administrative datasets to measure area-level deprivation. This represents a substantial methodological shift from the Census-based NZDep. Using our bespoke Data Zones (DZs), which are an intermediary boundary layer between Census Meshblocks and Area Units, we developed the New Zealand Index of Multiple Deprivation (IMD) comprising 28 indicators representing seven Domains of deprivation (Income, Employment, Crime, Housing, Health, Education and Geographical Access). DZs, small geographic areas with an average population of 712, were ranked by ascending levels of deprivation to provide a nuanced and flexible measure of multiple aspects of deprivation in New Zealand. The seven Domains of deprivation were weighted according to their theoretical ability to directly measure different forms of deprivation in New Zealand, how well the indicators in each Domain measured those forms of deprivation, and the quality and coverage of the data they used.

Noble et al[30] provided a thorough critical assessment of five potential approaches for defining the Domain weights used to construct an IMD. These include: considering the literature on social exclusion and material deprivation; calculating the weights empirically; defining the weights according to policy requirements; determining the weights by consensus; or entirely arbitrary. In this research, and consistent with existing IMDs, our weights were driven according to relevant literature. Whilst our weights are similar to those used in the UK indices of multiple deprivation for most Domains, we made minor adjustments to the Housing and Access Domains, in response to the New Zealand context. The Housing Domain was given a weight of 9% (it received 2% in the 2012 SIMD) because of the importance of adequate housing for health and social outcomes in NZ, [52] the persistence of overcrowding [53] as a manifestation of housing shortages, general declines in housing affordability and home ownership rates, [54] and the robustness of data for the two indicators which met our criteria. The Access Domain was given a weight of 2% (it received 9% in the 2012 SIMD) because unlike the UK, NZ studies have shown that access to basic services has a negative association with deprivation, except in rural areas. Pearce et al. [47] found that access to community resources is better in more deprived urban areas and best in intermediate urban/rural areas. However, for rural areas, access to the majority of resources is worse in more deprived areas.

A significant strength of the IMD is that the seven Domains may be used individually or in combination to explore the geography of deprivation and its association with a given health or social outcome. For example, a researcher wishing to investigate the association between household overcrowding and deprivation using the IMD can choose to exclude the Housing Domain from the overall index, or use combinations of Domains such as Employment, Income and Health.

In the UK, the inclusion of an Income Domain within an index of multiple deprivation is universal. In the 2012 SIMD for example, the Income Domain included the proportion of the population receiving different types of income support. [36] However, since the dollar amount paid to an individual may vary based on their circumstances and the value of the benefits they are eligible for, in this study we used the dollar amount paid in the form of Working for Families Tax Credit payments and selected income-tested benefits to measure the level of income deprivation in each DZ.

The Employment Domain comprises indicators representing the working age (15–64) population who were receiving the Unemployment Benefit or the Sickness Benefit in the last week of March 2013, as this was the Ministry of Social Development’s (MSD) reporting period closest to the 2013 Census night. As the MSD data are available in the Integrated Data Infrastructure, we will explore the potential to develop seasonally-adjusted Employment Domain indicators in future iterations of the IMD, consistent with the IMDs in the UK.[31]

By using data from routine administrative datasets we have mitigated problems associated with self-report bias that some national surveys may face, and made it possible to update the IMD regularly. A motivation to align the IMD with the 2013 Census data was to ensure a reliable population denominator was available. Recently, a methodology was proposed for constructing a denominator population within the IDI, based on an (anonymised) individual’s inclusion in key government databases (e.g. tax, health, vital statistics, and immigration), therefore updates at more frequent intervals may be possible using the denominators available from Statistics NZ’s IDI. [55] Future updates of the IMD will be conducted within the IDI environment to maximise the use of routine administrative data and regularly updated address information, geocoded to MBs, which can be easily aggregated to DZs. [56] Nevertheless, subsequent iterations of the IMD will need to be mindful of managing changing geographies, and variations in the collection and reporting of administrative datasets, as has occurred for successive iterations of the IMDs developed by the constituent countries of the UK.

Despite its benefits, the use of big datasets, such as those used to develop the IMD, does pose challenges. For some indicators it was difficult to assign counts to the correct DZ either because the residential address of the person was poorly recorded or the organisation providing the data was using out-dated geographical boundary maps. For example, the MSD mapped their clients’ addresses to the 2001 MBs, which are quite large and in many cases overlap more than one DZ. We developed a methodology to assign the MSD data to the correct DZ by calculating the proportion of the 2013 population in each part of the 2001 MB, and using those proportions to assign the MSD data to the relevant DZs. If a 2001 MB lay entirely within one of the 5,958 DZs, then 100% MSD data values of this MB were assigned to the corresponding DZ. If a 2001 MB overlapped two or more DZs, then the proportion of the 2013 population of each DZ was used to determine the percentage of MSD data values assigned to each DZ. This method assumed that every person was equally likely to receive MSD payments or benefits and was used for 2.21% (834/37,708) of the 2001 MBs.

Internationally, there is debate around whether shrinkage should be applied to the estimates used in the development of an IMD to deal with unreliable extreme estimates from areas with small populations. A number of shrinkage techniques have been proposed. [29, 30, 57–60] While the 2004 SIMD used shrinkage techniques, a subsequent evaluation of the statistical methods used in the development of the SIMD [57] concluded that shrinkage estimation was unnecessary. We contend that using national or DZ-level shrinkage techniques may mask small pockets of deprivation. [61] Furthermore, while Bayesian spatial shrinkage methods model the correlation between geographically proximate DZs, their effective use may be limited due to New Zealand’s elongated physical topography. Thus, shrinkage estimation was not applied in the development of the IMD.

Various Government agencies that provided data have expressed interest in using the IMD to investigate the geography of vaccination uptake, tertiary education participation and outcomes, demand for services among vulnerable children, and uptake of Working for Families Tax Credits. Furthermore, we are having ongoing discussions with Statistics New Zealand about the DZs, which may be used in their geographic boundary review. Given the interest in the DZs and the IMD from researchers and policy analysts, we foresee their potential in planning and funding. In the UK for example, Local Authorities that were eligible to receive a share of £1.5Billion between 2008/9 and 2010/11 from the Working Neighbourhoods Fund (WNF) were identified using the English IMD and its Employment Domain.[62] Similarly the National Health Service (NHS) uses the IMD as part of its weighted capitation funding modelling, which allocated over £85million to primary care trusts and deprivation-weighted top-ups to physician salaries in England in 2011/12.[63] We are confident that the adoption of our DZs will be similar to other intermediate geographies in the UK, which have been used widely in research, planning and government, rather than a tool to be used only by researchers, such as the Primary Care Service Areas developed as part of the Dartmouth Atlas of Healthcare Variation.[64]

Together, the DZs, the IMD and its Domains provide numerous opportunities for future research. In addition to the Government driven projects mentioned above, we have conducted a study demonstrating associations between childhood obesity and deprivation in which we outline the use of the overall IMD, its separate domains, and the IMD excluding the Health Domain. Suggestions for future research include using the five health indictors as outcomes against which the IMD can be tested to give more empiric weighting to the other domains and to explore geographical variations in domain ranks. We also plan to explore variations in CVD medication dispensing, proximity to fast food outlets, and further research on Māori/non-Māori inequities in health and social outcomes.

This paper provides an overview of the IMD and its development. We have developed an interactive online atlas, comprising the IMD and Domain ranks in addition to a selection of demographic data from the 2013 Census and a technical report for users to explore the IMD in more detail. Our atlas enables users to explore variations by District Health Board, Territorial Authority, General Electoral District, and Iwi boundaries. Furthermore, as part of our ongoing research, we are currently investigating the relationship between deprivation and a number of health and social outcomes, with a view to understanding service delivery gaps better, and facilitating the targeting of resources for health or social well-being. We are also exploring the associations between NZDep2013 and the IMD in terms of the geography of deprivation in New Zealand. All resources related to the IMD, its Domains, and the DZs are available from http://www.fmhs.auckland.ac.nz/imd.html

## Supporting information

S1 Fig The SIMD 2012 Methodology overview, in The Scottish Index of Multiple Deprivation 2012 Methodology Visual Guide. Edinburgh: Scottish Government (Crown copyright 2012).
(PNG)

S1 File Written Permission from the Scottish Government to adapt the visual description of the SIMD 2012 Methodology overview. (PDF)

## References

[1] Abas MA, Vanderpyl J, Robinson E, Le Prou T, Crampton P. Socio-economic deprivation and duration of hospital stay in severe mental disorder. British Journal of Psychiatry. 2006;188:581–2. doi: 10.1192/bjp.bp.104.007476 16738350 10.1192/bjp.bp.104.007476

[2] Atkinson JS, Clare; Crampton, P. NZDEP2013 Index of Deprivation. Wellington: 2014.

[3] Barnett R, Lauer G. Urban deprivation and public hospital admissions in Christchurch, New Zealand, 1990–1997. Health & Social Care in the Community. 2003;11(4):299–313. 14629201 10.1046/j.1365-2524.2003.00425.x

[4] Utter J, Denny S, Crengle S, Ameratunga S, Robinson E, Clark T, et al Overweight among New Zealand adolescents: associations with ethnicity and deprivation. International Journal of Pediatric Obesity. 2010;5(6):461–6. Epub 2010/03/18. doi: 10.3109/17477160903568439 . 20233145 10.3109/17477160903568439

[5] Butler DC, Petterson S, Phillips RL, Bazemore AW. Measures of Social Deprivation That Predict Health Care Access and Need within a Rational Area of Primary Care Service Delivery. Health Services Research. 2012:n/a-n/a. doi: 10.1111/j.1475-6773.2012.01449.x 22816561 10.1111/j.1475-6773.2012.01449.x PMC3626349

[6] Carstairs V, Morris R. Deprivation: explaining differences in mortality between Scotland and England and Wales. Bmj. 1989;299(6704):886–9. 2510878 10.1136/bmj.299.6704.886 PMC1837760

[7] Exeter DJ, Boyle PJ, Norman P. Deprivation (im)mobility and cause-specific premature mortality in Scotland. Social Science & Medicine. 2011;72(3):389–97. Epub 2010/11/26. doi: 10.1016/j.socscimed.2010.10.009 . 21106285 10.1016/j.socscimed.2010.10.009

[8] Kleinschmidt I, Hills M, Elliott P. Smoking behaviour can be predicted by neighbourhood deprivation measures. J Epidemiol Community Health. 1995;49 Suppl 2:S72–7. ; PubMed Central PMCID: PMC1060880. 8594138 10.1136/jech.49.suppl_2.s72 PMC1060880

[9] Noble M, Barnes H, Wright G, Roberts B. Small area indices of multiple deprivation in South Africa. Social indicators research. 2010;95(2):281–97.

[10] Norman P, Boyle P, Exeter D, Feng Z, Popham F. Rising premature mortality in the UK's persistently deprived areas: Only a Scottish phenomenon? Social Science and Medicine. 2011;73(11):1575–84. doi: 10.1016/j.socscimed.2011.09.034 22030211 10.1016/j.socscimed.2011.09.034

[11] Herbert DT. Urban deprivation: definition, measurement and spatial qualities. Geographical journal. 1975:362–72.

[12] Holtermann S. Areas of urban deprivation in Great Britain: an analysis of 1971 census data. Social Trends. 1975;6:33–47.

[13] Townsend P. Deprivation. Journal of social policy. 1987;16(2):125–46.

[14] Black D, Morris J, Smith C, Townsend P. The Black report: inequalities in health London: DHSS 1980.

[15] Department of the Environment Transport and the Regions. Updating and Revising the Index of Local Deprivation. Department of the Environment,Transport and the Regions, 1998.

[16] Reinken J, McLeod JW, Murphy TID. Health and Equity. Wellington, New Zealand: Department of Health, 1985.

[17] Salmond CE, Crampton P. Development of New Zealand's deprivation index (NZDep) and its uptake as a national policy tool. Canadian Journal of Public Health/Revue Canadienne de Sante'e Publique. 2012:S7–S11. 23618071

[18] Baker MG, Barnard LT, Kvalsvig A, Verrall A, Zhang J, Keall M, et al Increasing incidence of serious infectious diseases and inequalities in New Zealand: a national epidemiological study. Lancet. 2012;379(9821):1112–9. Epub 2012/02/23. doi: 10.1016/S0140-6736(11)61780-7 . 22353263 10.1016/S0140-6736(11)61780-7

[19] Norris P, Horsburgh S, Keown S, Arroll B, Lovelock K, Cumming J, et al Too much and too little? Prevalence and extent of antibiotic use in a New Zealand region. Journal of antimicrobial chemotherapy. 2011:dkr194. 10.1093/jac/dkr194 21622675

[20] Pearce J, Witten K, Hiscock R, Blakely T. Are socially disadvantaged neighbourhoods deprived of health-related community resources? International Journal of Epidemiology. 2007;36(2):348–55. doi: 10.1093/ije/dyl267 17182634 10.1093/ije/dyl267

[21] Utter J, Scragg R, Schaaf D. Associations between television viewing and consumption of commonly advertised foods among New Zealand children and young adolescents. Public health nutrition. 2006;9(05):606–12. 16923292 10.1079/phn2005899

[22] Wheeler BW, Rigby JE, Huriwai T. Pokies and poverty: problem gambling risk factor geography in New Zealand. Health & place. 2006;12(1):86–96. 16243683 10.1016/j.healthplace.2004.10.011

[23] Penno E, Gauld R, Audas R. How are population-based funding formulae for healthcare composed? A comparative analysis of seven models. BMC health services research. 2013;13(1):1. 24209410 10.1186/1472-6963-13-470 PMC4225752

[24] Tumen S. The impact of school resourcing and financial management on educational attainment and achievement: ResearchSpace@ Auckland; 2013.

[25] Callister P. Special measures to reduce ethnic disadvantage in New Zealand. Wellington: Institute of Policy Studies, Victoria University 2007.

[26] Christie SML, Fone DL. Does car ownership reflect socio-economic disadvantage in rural areas? A cross-sectional geographical study in Wales, UK. Public Health. 2003;117(2):112–6. doi: 10.1016/S0033-3506(02)00027-6 12802977 10.1016/S0033-3506(02)00027-6

[27] Ministry of Social Development. The Social Report 2010 Wellington: Ministry of Social Development, 2010.

[28] Atkinson J, Salmond C, Crampton P. NZDep2013 Index of Deprivation. 2014.

[29] Noble M, Penhale B, Smith G, Wright G, Dibben C, Owen T, et al Indices of Deprivation 2000. Regeneration Research Summary, Number 31,. Department of Transport, Environment and the Regions (DETR), 2000.

[30] Noble M, Wright G, Smith G, Dibben C. Measuring multiple deprivation at the small-area level. Environment and Planning A. 2006;38(1):169.

[31] Smith T, Noble M, Noble S, Wright G, McLennan D, Plunkett E. The English Indices of Deprivation 2015: Technical Report. London: Department for Communities and Local Government, https://www.gov.uk/government/uploads/system/uploads/attachment_data/file/464485/English_Indices_of_Deprivation_2015_-_Technical-Report.pdf, 2015.

[32] Zhao J, Exeter DJ. Developing intermediate zones for analysing the social geography of Auckland, New Zealand. New Zealand Geographer. 2016;72(1):14–27.

[33] Exeter DJ, Zhao J, Browne M, Lee AC. Towards a new Index of Multiple Area‐Level Deprivation for Auckland, New Zealand. New Zealand Geographer. 2016.

[34] Pampalon R, Hamel D, Gamache P, Raymond G. A deprivation index for health planning in Canada. Chronic diseases in Canada. 2009;29(4):178–91. Epub 2009/10/07. . 19804682

[35] Crampton P, Salmond C, Sutton F. *NZDep91*: Index of Deprivation. Wellington: Health Services Research Centre, 1997.

[36] Scottish Government. Scottish Index of Multiple Deprivation 2012:Methodology Visual Guide. Available at: http://www.gov.scot/Topics/Statistics/SIMD/BackgroundMethodology/MethodologyVisual2012. Last Accessed 11 May 2017. Edinburgh: Scottish Government, 2012.

[37] Statistics New Zealand. Introduction to the Integrated Data Infrastructure 2013. 2013.

[38] Statistics New Zealand. Recorded crime victims statistics–release notes by date: Statistics NZ; 2016 [updated 1st June 2016; cited 2016 26 July 2016]. Release notes by date]. Available from: http://www.stats.govt.nz/tools_and_services/nzdotstat/tables-by-subject/recorded-crime-victims/recorded-crime-victim-release-notes.aspx.

[39] Canada Mortgage and Housing Corporation. 2011 Census/National Household Survey Housing Conditions Series: Issue 2 The Geography of Core Housing Need in 2011. Canada: CMHC, 2014.

[40] Northern Ireland Statistics and Research Agency. Northern Ireland Multiple Deprivation Measure 2010. Belfast: Northern Ireland Statistics and Research Agency; 2010.

[41] Government W. Welsh Index of Multiple Deprivation. Cardiff: Office for National Statistics, 2014.

[42] DETR. Indices of Deprivation. London: Department of the Environment, Transport and the Regions, 2000.

[43] Blakely T, Ajwani S, Robson B, Tobias M, Bonne M. Decades of disparity: widening ethnic mortality gaps from 1980 to 1999. The New Zealand medical journal. 2004;117(1199):U995 . 15475978

[44] Alford-Teaster J, Lange JM, Hubbard RA, Lee CI, Haas JS, Shi X, et al Is the closest facility the one actually used? An assessment of travel time estimation based on mammography facilities. International Journal of Health Geographics. 2016;15(1):1–10. doi: 10.1186/s12942-016-0039-7 26892310 10.1186/s12942-016-0039-7 PMC4757990

[45] Langford M, Higgs G. Accessibility and public service provision: evaluating the impacts of the Post Office Network Change Programme in the UK. Transactions of the Institute of British Geographers. 2010;35(4):585–601.

[46] Exeter DJ, Zhao J, Browne M, Lee AC. Towards a new Index of Multiple Area‐Level Deprivation for Auckland, New Zealand. New Zealand Geographer. 2016;72(2):92–106.

[47] Pearce J, Witten K, Hiscock R, Blakely T. Regional and urban-rural variations in the association of neighbourhood deprivation with community resource access: a national study. Environment and planning A. 2008;40(10):2469.

[48] Zealand SN. Classification of Urban Area. Available at: http://www.stats.govt.nz/methods/classifications-and-standards/classification-related-stats-standards/urban-area.aspx. Wellington: Statistics New Zealand, 2013.

[49] Milne BJ. New Zealand Socio-economic Index 2006 (NZSEI-06): An introduction for social science researchers. New Zealand Sociology. 2012;27(2):117.

[50] Jensen J. Income Equivalences and the Estimation of Family Expenditures on Children. Wellington: Department of Social Welfare, 1988.

[51] Carter K, Gunasekara F, Blakely T. The relationship between trends in income inequalities and poverty in New Zealand. Policy Quarterly. 2013;9(2):24–9.

[52] NZCPHM. New Zealand College of Public Health Medicine Policy Statement New Zealand College of Public Health Medicine, 2013.

[53] CPAG. Room for Improvement. Current New Zealand housing policies and their implications for our children. 2003.

[54] NZPC. Housing Affordability inquiry. Summary version. New Zealand Productivity Commission, 2012.

[55] Gibb S, Bycroft C, Matheson-Dunning N. Identifying the New Zealand resident population in the Integrated Data Infrastructure (IDI). Retrieved from www.stats.govt.nz. Wellington: Statistics New Zealand, 2016.

[56] Statistics NZ. Data in the IDI 2016 [27 July 2016]. List of microdata available in the IDI, including the data dictionaries for each collection]. Available from: http://www.stats.govt.nz/browse_for_stats/snapshots-of-nz/integrated-data-infrastructure/idi-data.aspx.

[57] McConnachie A, Weir C. Evaluation of Statistical Techniques in the Scottish Index of Multiple Deprivation,. Edinburgh:: The Scottish Executive, http://www.scotland.gov.uk/Publications/2005/10/1893201/32023, 2005.

[58] Besag J, York J, Mollié A. Bayesian image restoration, with two applications in spatial statistics. Ann Inst Stat Math. 1991;43(1):1–20. doi: 10.1007/BF00116466

[59] Datta G, Ghosh M. Small Area Shrinkage Estimation. 2012:95–114. doi: 10.1214/11-STS374

[60] Longford NT. Multivariate shrinkage estimation of small area means and proportions. Journal of the Royal Statistical Society: Series A (Statistics in Society). 1999;162(2):227–45. doi: 10.1111/1467-985X.00132.

[61] Exeter D, Flowerdew R, Boyle P. Policy Implications of Pockets of Deprivation in Scotland In: Wise S, Craglia M, editors. GIS and Evidence-Based Policy Making. Boca Raton, Florida, USA: CRC Press; 2008 p. 95–112.

[62] Department for Communities and Local Government. Working Neighbourhood Fund allocations. London, UK: Department for Communities and Local Government, 2008.

[63] Department of Health. Resource Allocation: Weighted Capitation Formula, Seventh Edition London, UK: Department of Health, 2011.

[64] Goodman DC, Mick SS, Bott D, Stukel T, Chang CH, Marth N, et al Primary care service areas: a new tool for the evaluation of primary care services. Health Serv Res. 2003;38(1 Pt 1):287–309. doi: 10.1111/1475-6773.00116 ; PubMed Central PMCID: PMC1360885. 12650392 10.1111/1475-6773.00116 PMC1360885

