# Supplementary figures and images for: The New Zealand Indices of Multiple Deprivation (IMD): A new suite of indicators for social and health research in Aotearoa, New Zealand
Source: PLoS One. 2017 Aug 3;12(8):e0181260. doi: 10.1371/journal.pone.0181260 (PMC5542612; doi:10.1371/journal.pone.0181260)

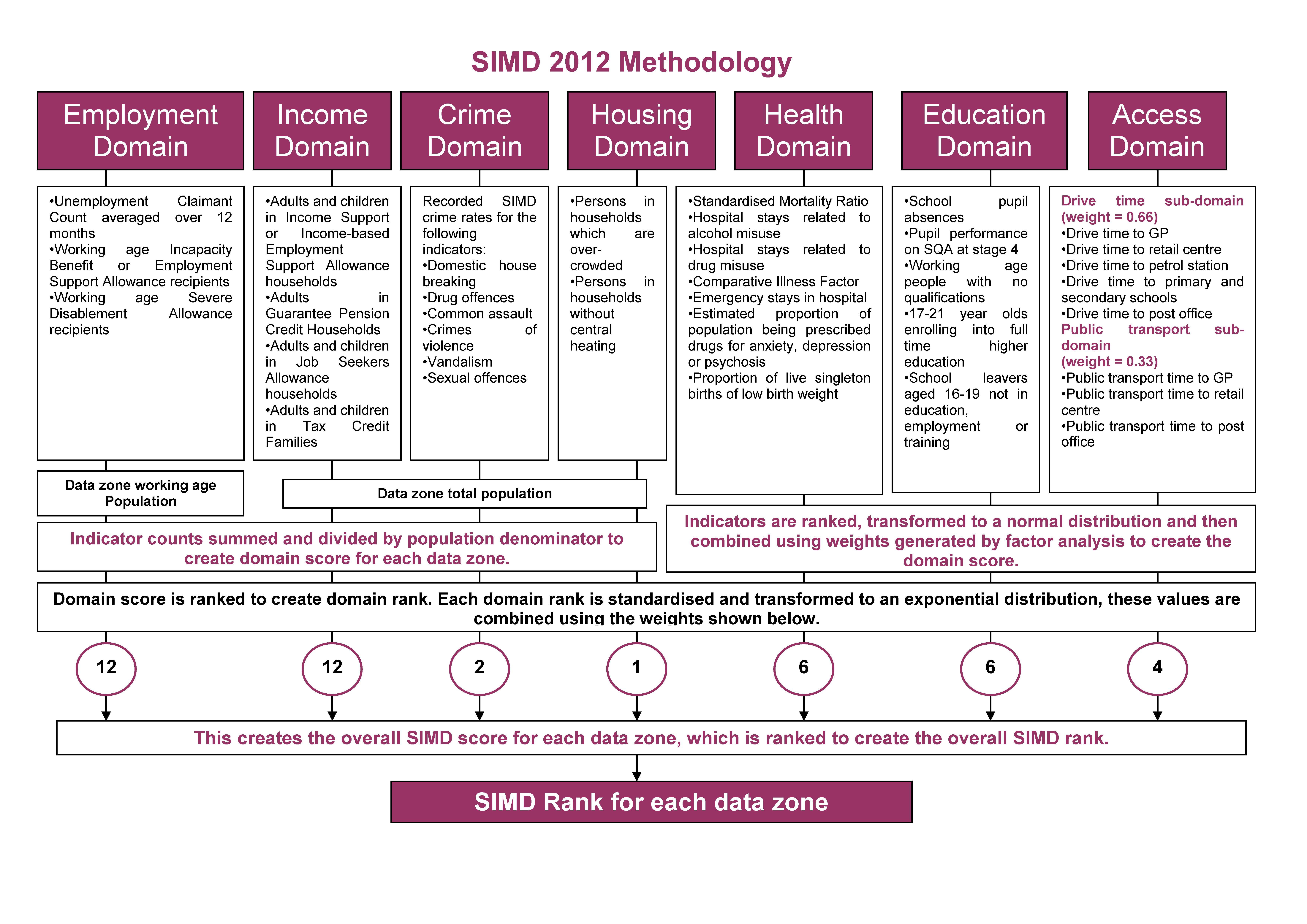

Supplement: S1 Fig — Edinburgh: Scottish Government (Crown copyright 2012). (PNG) [file pone.0181260.s001.png]
