# Supplementary material for: The New Zealand Indices of Multiple Deprivation (IMD): A new suite of indicators for social and health research in Aotearoa, New Zealand
Source: PLoS One. 2017 Aug 3;12(8):e0181260. doi: 10.1371/journal.pone.0181260 (PMC5542612; doi:10.1371/journal.pone.0181260)
Supplement: S1 File — (PDF) [file pone.0181260.s002.pdf]

Housing and Social Justice Directorate
Communities Analysis Division

Scottish Government
Riaghaltas na h-Alba
gov.scot

T: 0131-244 7714
E: alastair.mcalpine@gov.scot

Alastair McAlpine
Scottish Government

Victoria Quay
Leith
Edinburgh
EH6 6QQ

2nd May 2017

Dear Sir/Madam

Following a request from University of Auckland to reproduce material published for the Scottish Index of Multiple Deprivation (SIMD), for the purposes of the Index of Multiple Deprivation (IMD) for New Zealand, specifically permission to publish a version of the SIMD 2012 Methodology diagram under a CC BY license for the IMD for New Zealand.

I confirm that this proposal is fair use of SIMD material and can therefore be re-used for the purpose of the IMD for New Zealand.

The SIMD is published by the Scottish Government and protected by Crown Copyright. The terms and conditions of this copyright are published under the 'Open Government License' (http://www.nationalarchives.gov.uk/doc/open-government-licence/version/3/)

Under this license you are free to re-use the Information in SIMD for commercial or non-commercial purposes with reference to the original sources.

If you need any further assistance then please contact me on the details above.

Alastair McAlpine
Statistician
Scottish Index of Multiple Deprivation (SIMD) team
Equalities, Poverty and Social Justice
Communties Analysis Division

From: Alastair.McAlpine@gov.scot
To: Dan Exeter (EpiBio)
Subject: RE: Request for the SIMD to grant a Creative Commons Attribution License
Date: Thursday, 27 April 2017 3:30:31 a.m.

Dan,

I can confirm that you are free to use the material as detailed in your email.

The SIMD is published by the Scottish Government and protected by Crown Copyright. The terms and conditions of this copyright are published under the 'Open Government License' which is available to view here:
http://www.nationalarchives.gov.uk/doc/open-government-licence/version/3/

I draw your attention to the following conditions:

"You are free to:
- copy, publish, distribute and transmit the Information;
- adapt the Information;
- exploit the Information commercially and non-commercially for example, by combining it with other Information, or by including it in your own product or application."

"acknowledge the source of the Information in your product or application by including or linking to any attribution statement specified by the Information Provider(s) and, where possible, provide a link to this licence;"

I hope this helps.

Alastair McAlpine
Tel: (0131 24)4 7714
Mob: 07580 668 305
Latest SIMD newsletter (pdf)

From: Dan Exeter (EpiBio) [mailto:d.exeter@auckland.ac.nz]
Sent: 26 April 2017 05:22
To: McAlpine A (Alastair)
Cc: Dan Exeter (EpiBio)
Subject: Request for the SIMD to grant a Creative Commons Attribution License
Importance: High

Dear Alastair

As you know, we have developed an Index of Multiple Deprivation (IMD) for New Zealand following the methodology developed for the 2012 Scottish IMD. We have submitted an article for publication in the PLOS ONE journal. Our article includes a diagram (see below) that follows the style of the diagram labelled 'Figure 4.2: SIMD 2012 Methodology' published in **The Scottish Index of Multiple Deprivation 2012 Visual Guide**

Currently the diagram includes the caption that appears below, but the journal's policy requires a Creative Commons Attribution License.

I request permission for the open-access journal PLOS ONE to publish the New Zealand version of the SIMD 2012 Methodology diagram under the Creative Commons Attribution License (CCAL) CC BY 4.0 (http://creativecommons.org/licenses/by/4.0/). Please be aware that this license allows unrestricted use and distribution, even commercially, by third parties.

Please reply and provide explicit written permission to publish the New Zealand version of the SIMD 2012 Methodology diagram under a CC BY license.

Here is the New Zealand version of the SIMD 2012 Methodology diagram:

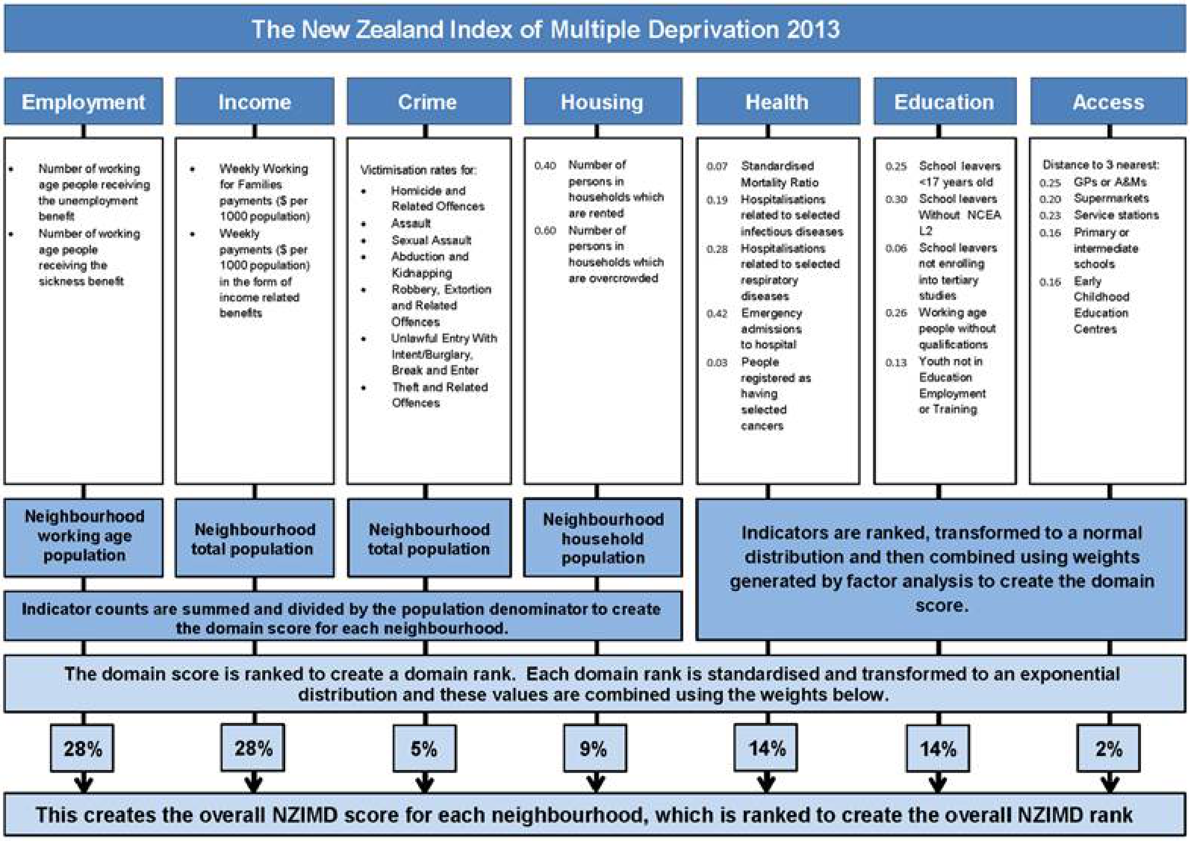

Figure 1. Developing the NZ Indices of Multiple Deprivation: An overview of indicators, domains and weights. Adapted from Figure 4.2 SIMD 2012 Methodology, in **The Scottish Index of Multiple Deprivation 2012 Visual Guide**[1]. Edinburgh: Scottish Government (Crown copyright 2012).

1. The Scottish Government. Scottish index of multiple deprivation 2012 http://www.gov.scot/Topics/Statistics/SIMD/BackgroundMethodology/MethodologyVisual2012. Last Accessed 26 April 2017

We appreciate all the support and advice you have already provided, and look forward to hearing back from you soon.

Regards,

Dr Daniel Exeter

Epidemiology & Biostatistics,
School of Population Health
University of Auckland
d.exeter@auckland.ac.nz
MOB: +64 21 436 603

____
This email has been scanned by the Symantec Email Security.cloud service.
For more information please visit http://www.symanteccloud.com
____

*********************************** ********************************

This email has been received from an external party and

has been swept for the presence of computer viruses.

****

****

This e-mail (and any files or other attachments transmitted with it) is intended solely for the attention of the addressee(s). Unauthorised use, disclosure, storage, copying or distribution of any part of this e-mail is not permitted. If you are not the intended recipient please destroy the email, remove any copies from your system and inform the sender immediately by return.

Communications with the Scottish Government may be monitored or recorded in order to secure the effective operation of the system and for other lawful purposes. The views or opinions contained within this e-mail may not necessarily reflect those of the Scottish Government.

Tha am post-d seo (agus faidhle neo ceanglan còmhla ris) dhan neach neo luchd-ainmichte a-mhàin. Chan eil e ceadaichte a chleachdadh ann an dòigh sam bith, a’ toirt a-steach còraichean, foillseachadh neo sgaoileadh, gun chead. Ma ’s e is gun d’fhuair sibh seo le gun fhiosd’, bu choir cur às dhan phost-d agus lethbhreac sam bith air an t-siostam agaibh, leig fios chun neach a sgaoil am post-d gun dàil.

Dh’fhaodadh gum bi teachdaireachd sam bith bho Riaghaltas na h-Alba air a chlàradh neo air a sgrùdadh airson dearbhadh gu bheil an siostam ag obair gu h-èifeachdach neo airson adhbhar laghail eile. Dh’fhaodadh nach eil beachdan anns a’ phost-d seo co-ionann ri beachdan Riaghaltas na h-Alba.

****
